# The Effect of Increasing State Minimum Wage on Family Caregiving

**DOI:** 10.1093/geroni/igab046.1930

**Published:** 2021-12-17

**Authors:** Eric Jutkowitz, Derek Lake, Peter Shewmaker

**Affiliations:** 1 Brown University, Brown University, Rhode Island, United States; 2 Brown Universtiy, Brown University, Rhode Island, United States

## Abstract

Paid long-term care workers, such as personal care aides, compliment family caregivers in the delivery of care for people with long-term care needs. Nearly 12% of paid long-term care workers live in poverty. There is a call to raise the federal minimum wage from $7.25/hr to $15/hr. Long-term care workers may financially benefit from an increase in minimum wage, but families that rely on paid long-term care may be unable to afford higher wages. We obtained Health and Retirement Study (HRS; 2006-2014) respondents’ state of residence which we linked with state minimum wage data. Between 2006 and 2010 the federal minimum wage increased from $5.15 to $7.25. We identified 25 states in which the 2006 to 2010 (pre period) increases in federal minimum wage increased the state’s effective minimum wage (higher of state and federal minimum wage). Seven of these states continued to increase their minimum wage from 2010 to 2014 (post period). The remaining 18 matching control states did not increase their minimum wage after 2010. We used a difference-in-differences design and ordinary least squares regression to compare hours of unpaid and paid caregiving HRS respondents received in treatment and control. There was no statistically significant change in unpaid (-2.15; 95%CI: -8.53, 4.23) or paid (2.42; 95%CI: -1.33, 6.20) caregiving hours received between HRS respondents that lived in states that did and did not increase their minimum wage. Increasing state minimum wage may improve the economic wellbeing of long-term care workers without adversely affecting people with long-term care needs.

